# The prognostic value of *KRAS* mutation by cell-free DNA in cancer patients: A systematic review and meta-analysis

**DOI:** 10.1371/journal.pone.0182562

**Published:** 2017-08-10

**Authors:** Rongyuan Zhuang, Song Li, Qian Li, Xi Guo, Feng Shen, Hong Sun, Tianshu Liu

**Affiliations:** 1 Department of Medical Oncology, Zhongshan Hospital, Fudan University, Shanghai, China; 2 Department of Surgery, Affiliated Tumor Hospital of Anhui University of Science & Technology (Huainan City Dongfang Tumor Hospital), Huainan, Anhui Province, China; 3 Department of Clinical Pharmacy, School of Pharmacy, Fudan University, Shanghai, China; CNR, ITALY

## Abstract

*KRAS* mutation has been found in various types of cancer. However, the prognostic value of *KRAS* mutation in cell-free DNA (cfDNA) in cancer patients was conflicting. In the present study, a meta-analysis was conducted to clarify its prognostic significance. Literature searches of Cochrane Library, EMBASE, PubMed and Web of Science were performed to identify studies related to *KRAS* mutation detected by cfDNA and survival in cancer patients. Two evaluators reviewed and extracted the information independently. Review Manager 5.3 software was used to perform the statistical analysis. Thirty studies were included in the present meta-analysis. Our analysis showed that *KRAS* mutation in cfDNA was associated with a poorer survival in cancer patients for overall survival (OS, HR 2.02, 95% CI 1.63–2.51, *P*<0.01) and progression-free survival (PFS, HR 1.64, 95% CI 1.27–2.13, *P*<0.01). In subgroup analyses, *KRAS* mutation in pancreatic cancer, colorectal cancer, non-small cell lung cancer and ovarian epithelial cancer had HRs of 2.81 (95% CI 1.83–4.30, *P*<0.01), 1.67 (95% CI 1.25–2.42, *P*<0.01), 1.64 (95% CI 1.13–2.39, *P* = 0.01) and 2.17 (95% 1.12–4.21, *p* = 0.02) for OS, respectively. In addition, the ethnicity didn’t influence the prognostic value of *KRAS* mutation in cfDNA in cancer patients (*p* = 0.39). Prognostic value of KRAS mutation was slightly higher in plasma than in serum (HR 2.13 vs 1.65), but no difference was observed (*p* = 0.37). Briefly, *KRAS* mutation in cfDNA was a survival prognostic biomarker in cancer patients. Its prognostic value was different in various types of cancer.

## Introduction

In recent years, the molecular biomarkers are increasingly being regarded as both predictive and prognostic tools for cancer patients. Currently, the alterations detection in biomarkers is considered the standard of care in many types of cancer, including lung, pancreatic, and colorectal cancer. For example, the National Comprehensive Cancer Network (NCCN) guidelines recommend testing for the *KRAS* alterations as a part of the initial diagnostic check for metastatic colorectal cancer (CRC) [1].

*KRAS*, which is known as an important member of *RAS* family and encoded by the *KRAS* gene, is a small GTPase which cycles between active guanosine triphosphate (GTP)-bound (*KRAS-*GTP) and inactive guanosine diphosphate (GDP)-bound (*KRAS*-GDP) conformations. It plays a critical important role in normal tissue signaling. *KRAS* mutation can impair the intrinsic GTPase activity and lead to the permanent activation of its downstream signaling pathways, such as PI3K/AKT/mTOR and RAF/MEK/ERK [2,3]. Several studies have reported that *KRAS* mutation could enhance the cellular proliferation, induce the malignant transformation [4–6]. As a result, the continuous activation would contribute to the development and maintenance in cancer.

A growing number of studies indicated that *KRAS* mutation was a prognostic biomarker to predict the survival outcomes in cancer patients. A previous meta-analysis had suggested that *KRAS* mutation was associated with a poorer overall survival in patients with pancreatic cancer, especially when the mutation detection was performed by the circulating tumor DNA [7]. However, the prognostic value of *KRAS* mutation detected by cfDNA on survival in other cancer patients is still not completely clear. Thus, in the present study, we conducted a meta-analysis to investigate the effect of *KRAS* mutation detected by cfDNA on survival in patients with cancer.

## Methods

### Data sources and search strategy

The literature searches of EMBASE databases, Cochrane Library databases, and Web of Science were performed on June, 2016 and PubMed performed on March 2017. The main keywords used for the search were *K-ras* or *KRAS* or kirsten-ras or Kirsten ras or ki-ras, neoplasm or cancer or tumor or tumour or other subtypes/synonyms for cancer, liquid biopsy or serum or plasma or cell-free DNA or cell-free plasma DNA or cfDNA, and prognosis or survival. The detailed search terms and strategies were shown in S1 Table. Additionally, the full articles published were limited to English-language. The citation lists of retrieved articles were manually screened independently by two authors (ZYR and LS). All selected studies were checked according to a Newcastle-Ottawa Quality assessment Scale which was developed previously [8].

### Selection criteria

The inclusion criteria of our meta-analysis was as follows: (1) independently published observational study (case–control or cohort study) investigating the association between *KRAS* mutation detected by liquid biopsy and survival in cancer patients; (2) a study had reported the HR and its 95% CI for the association between *KRAS* mutation detected by cfDNA and survival in cancer patients; (3) a study had reported other indexes which could be used to calculate the HR and its 95% CI according to previously published methods[9,10]. In addition, the following exclusion criteria were also used: (1) abstracts and reviews; (2) studies without enough information; and (3) repeated or overlapping publications.

### Data extraction and quality assessment

The data extraction and quality assessment were performed by two investigators independently. The detailed information (first author, year of publication, period of study, the age of study population, country of study, ethnicity, cancer types and HR estimates) of each eligible study was collected. If several publications were overlapped, we selected the most recently published study or study with the largest numbers of subjects to be further analyzed. In addition, the discrepancies were reviewed and resolved in the present a third author (mainly SH).

The nine-star Newcastle–Ottawa Scale (NOS) was performed to assess the quality of each eligible study. With a NOS score equal or greater than seven, a study would be considered to be with high quality. An investigator would examine and adjudicate the information independently after data extraction and assessment.

### Statistical analysis

The HR and its related 95% CI reported or obtained by calculating in each study were performed to estimate the association between *KRAS* mutation in cfDNA and survival in cancer patients. If there was no heterogeneity existed, the fixed effects model was choose to assess the pooled HRs and its related 95%CIs; otherwise, the random effects model would be selected. The *Chi*^*2*^ and *I*^*2*^ statistic was used to assess and present the heterogeneity between the eligible studies. The funnel plot and Egger’s test were performed to assess the potential publication bias [11,12]. We considered that no publication bias existed, if the shape of the funnel plot was symmetrical and the *P* value of the Egger’s test was more than 0.05. In addition, a HR<1 indicated *KRAS* mutation was associated with a better outcome while HR>1 indicated *KRAS* mutation was associated with a worse outcome. *P* values were two sided and less than 0.05 were considered statistically different. The meta-analysis was performed through the Review Manager 5.3 software (Cochrane Collaboration).

## Results

### Literature search and study selection

**Fig 1:**
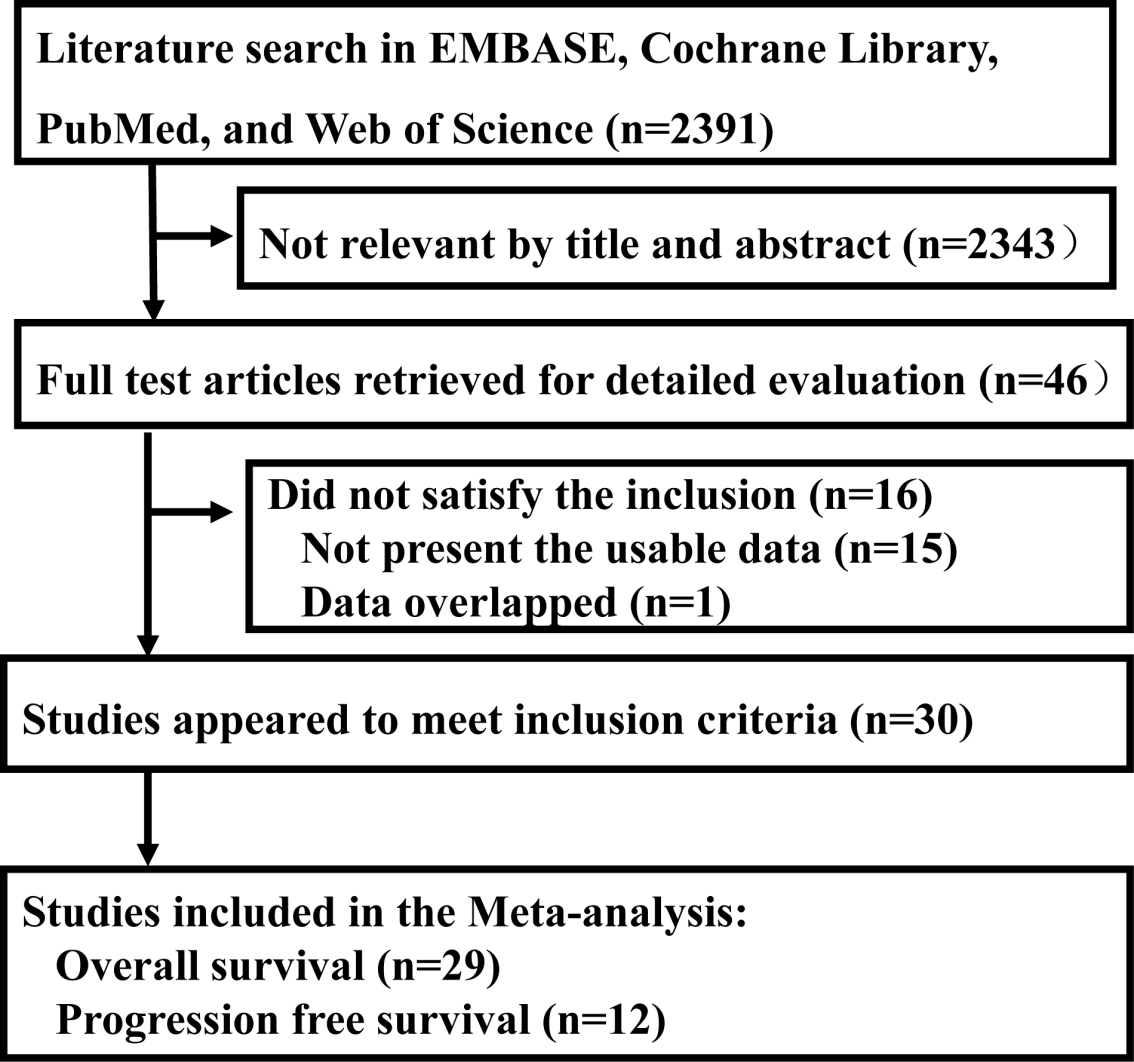
Flow chart of selection process for the eligible studies.

The literature searches resulted in 2391 studies at first. Then, 2343 records were excluded because of the duplications or no information on *KRAS* mutation detected by cfDNA and survival in cancer patients through the screening of the titles and abstracts of all studies. The rest of 46 records were screened by full texts. At last, there were 30 studies included in our meta-analysis [13–43]. The selection process for the eligible studies was shown in Fig 1.The main characteristics of the eligible studies were summarized in Table 1. In addition, quality assessment of the eligible studies was shown in S1 Table.

**Table 1:** The main characteristic of the studies included in the meta-analysis.

^Study^	^Country^	^Study Period^	^Age (years)^	^Tumor Types^	^Stage^	^KRAS mutation/Total^	^Detection methods^	^Outcomes^	^HR estimates^
^Camps,2005[^^14^^]^	^Spain^	^1999–2002^	^Median 64^	^Non-small cell lung cancer^	^IIIB-IV^	^20/67^	^Serum PCR-RFLP^	^OS, PFS^	^OS-KM^
^Camps,2011[^^13^^]^	^Spain^	^NA^	^Median 60^	^Non-small cell lung cancer^	^IIIB-IV^	^27/251^	^Plasma Allelic Discrimination with RT-PCR^	^OS, PFS^	^KM^
^Castells,1999[^^15^^]^	^Spain^	^1996–1997^	^Mean 62.6^	^Pancreatic cancer^	^I–IV^	^12/44^	^Plasma RELP-PCR^	^OS^	^KM^
^Chen,2010[^^16^^]^	^China^	^2007–2008^	^Median 60^	^Pancreatic cancer^	^Ⅲ–IV^	^30/91^	^Plasma Sequence^	^OS^	^HR+CI (m)^
^Dobrzycka,2011[^^17^^]^	^Poland^	^2002–2005^	^Median 58.3^	^Ovarian epithelial cancer^	^I–IV^	^27/126^	^Plasma PCR-RFLP^	^OS^	^KM^
^Earl,2015[^^18^^]^	^Spain^	^2009–2014^	^Median 68^	^Pancreatic cancer^	^LA, IV^	^8/31^	^Plasma ddPCR^	^OS^	^HR+P^
^El Messaoudi, 2016[^^19^^]^	^France^	^2010–2012^	^Median 66.6^	^Colorectal Cancer^	^IV^	^38/91^	^Plasma AS-PCR^	^OS^	^HR+CI^
^Gautschi,2007[^^20^^]^	^Switzerland^	^2001–2003^	^Median 61^	^Lung cancer^	^I–IV^	^16/175^	^Plasma PCR-RFLP^	^OS^	^HR+CI^
^Hadano,2016[^^21^^]^	^Japan^	^2007–2013^	^Median 69^	^Pancreatic cancer^	^I–IV^	^86/105^	^Plasma ddPCR^	^OS^	^KM^
^Han,2016[^^22^^]^	^Korea^	^NA^	^Median 58^	^non-small cell lung cancer^	^IIIB- IV^	^19/135 (OS) 7/59 (PFS)^	^Plasma PNA-PCR^	^OS, PFS^	^KM^
^Hara,2017[^^23^^]^	^Japan^	^2010–2013^	^Median 67^	^colorectal cancer^	^I-III^	^26/71^	^Plasma NA^	^OS, PFS^	^OS-KM^ ^PFS-HR+CI(m)^
^Janowski,2017[^^24^^]^	^United States^	^2011–2015^	^Median 56^	^colorectal cancer^	^IV^	^27/49^	^Plasma qPCR^	^OS^	^HR+CI(m)^
^Kim,2015[^^25^^]^	^Korea^	^2008–2011^	^Median 62^	^Colorectal Cancer^	^Advanced^	^26/65^	^Serum RFLP-PCR^	^OS^	^KM^
^Kimura,2004[^^26^^]^	^United States^	^2000–2002^	^Median 63^	^Non–Small-Cell Lung Cancer^	^IIIB-IV^	^5/25^	^Plasma RFLP-PCR^	^OS^	^KM^
^Kingham,2016[^^30^^]^	^United States^	^1990 to 2014^	^Age 59^	^Colorectal Cancer^	^I–IV^	^15/43^	^Serum qRT-PCR^	^OS^	^Survival rate^
^Kinugasa,2015[^^27^^]^	^Japan^	^2008–2010,^ ^2011–2013^	^Median 66^	^Pancreatic cancer^	^I–IV^	^101/141^	^Serum ddPCR-PHFA^	^OS^	^HR+CI^
^Laethem,2017[^^40^^]^	^German^	^NA^	^Median 63^	^Pancreatic cancer^	^II-IV^	^39/60^	^Plasma BEAMing^	^OS^	^HR+CI^
^Nygaard,2013[^^28^^]^	^Denmark^	^2007–2010^	^Median 66^	^Non-small cell lung cancer^	^Ⅱ-IV^	^43/246^	^Plasma ARMS-qPCR^	^OS, PFS^	^HR+CI(m)^
^Nygaard,2014[^^29^^]^	^Denmark^	^NA^	^Median 64^	^Non-small cell lung cancer^	^III-IV^	^7/58^	^Plasma ARMS-qPCR^	^OS, PFS^	^HR+CI^
^Ramirez,2003[^^31^^]^	^Spain^	^1998–1999^	^Median 62^	^Non-small cell lung cancer^	^I–IV^	^9/50^	^Serum RFLP-PCR^	^OS^	^KM^
^Semrad,2015[^^32^^]^	^United States^	^2009–2012^	^Median 67^	^Pancreatic cancer^	^Advanced or IV^	^10/27^	^Plasma ARMS^	^OS, PFS^	^KM^
^Singh,2015[^^33^^]^	^India^	^2007–2011^	^Mean 55^	^Pancreatic cancer^	^42% of IV^	^34/110^	^Plasma RFLP-PCR^	^OS^	^HR+CI^
^Spindler,2014[^^36^^]^	^Denmark^	^2010–2012^	^Median 62^	^Colorectal Cancer^	^IV^	^29/86^	^Plasma ARMS-qPCR^	^OS, PFS^	^HR+CI(m)^
^Spindler,2015[^^35^^]^	^Denmark^	^2010–2013^	^Median 63^	^Colorectal Cancer^	^IV^	^30/140^	^Plasma AS-PCR^	^OS, PFS^	^HR+CI (m)^
^Tabernero,2015[^^37^^]^	^Spain^	^2010–2011^	^Median 61^	^Colorectal Cancer^	^IV^	^349/503^	^Plasma BEAMing^	^OS, PFS^	^HR+P^
^Takai,2015[^^38^^]^	^Japan^	^2011–2014^	^Median 66^	^Pancreatic cancer^	^I–IV^	^83/259^	^Plasma ddPCR^	^OS^	^HR+CI (m)^
^Tjensvoll,2016[^^39^^]^	^Norway^	^2012–2014^	^Median 64^	^Pancreatic cancer^	^Advanced^	^10/14^	^Plasma ddPCR^	^OS, PFS^	^HR+P^
^Wang,2010[^^41^^]^	^China^	^2005–2008^	^>60 (53.8%)^	^non-small cell lung cancer^	^IIIB or stage IV^	^35/273^	^Plasma RFLP-PCR^	^PFS^	^KM^
^Xu,2014[^^42^^]^	^China^	^2007–2011^	^Median 56^	^Colorectal cancer^	^IV^	^76/242^	^Plasma PNA-PCR^	^OS^	^HR+CI (m)^
^Yamada,1998[^^43^^]^	^Japan^	^1994–1997^	^Mean 63.9^	^Pancreatic cancer^	^I–IV^	^11/15^	^Plasma MASA-PCR^	^OS^	^OS value^

HR, hazard ratio; CI, confidential interval; KM, Kaplan–Meier curve; AS-PCR, Allele-specific real-time quantitative PCR; m, multivariate analysis; p, p value

Among thirty included studies, 12 studies and 29 studies which reported the association of *KRAS* mutation detected by cfDNA with OS and PFS in cancer patients respectively. The cancer types of the eligible studies included pancreatic cancer, colorectal cancer, non-small cell lung cancer and ovarian epithelial cancer. Among 29 studies reporting OS, there were 10 studies focusing on Asian population and 19 studies on non-Asian population. Serum samples and plasma samples were used to detect *KRAS* mutation in 4 studies and 25 studies respectively.

### Qualitative assessment

The quality assessment of studies was shown in S2 Table. The scores of the eligible studies ranged from 6 to 8. The average NOS score of the eligible studies was 7.2 which indicating that most of the studies were with a high quality.

### Survival prognosis of *KRAS* mutation in cfDNA in cancer patients

**Fig 2:**
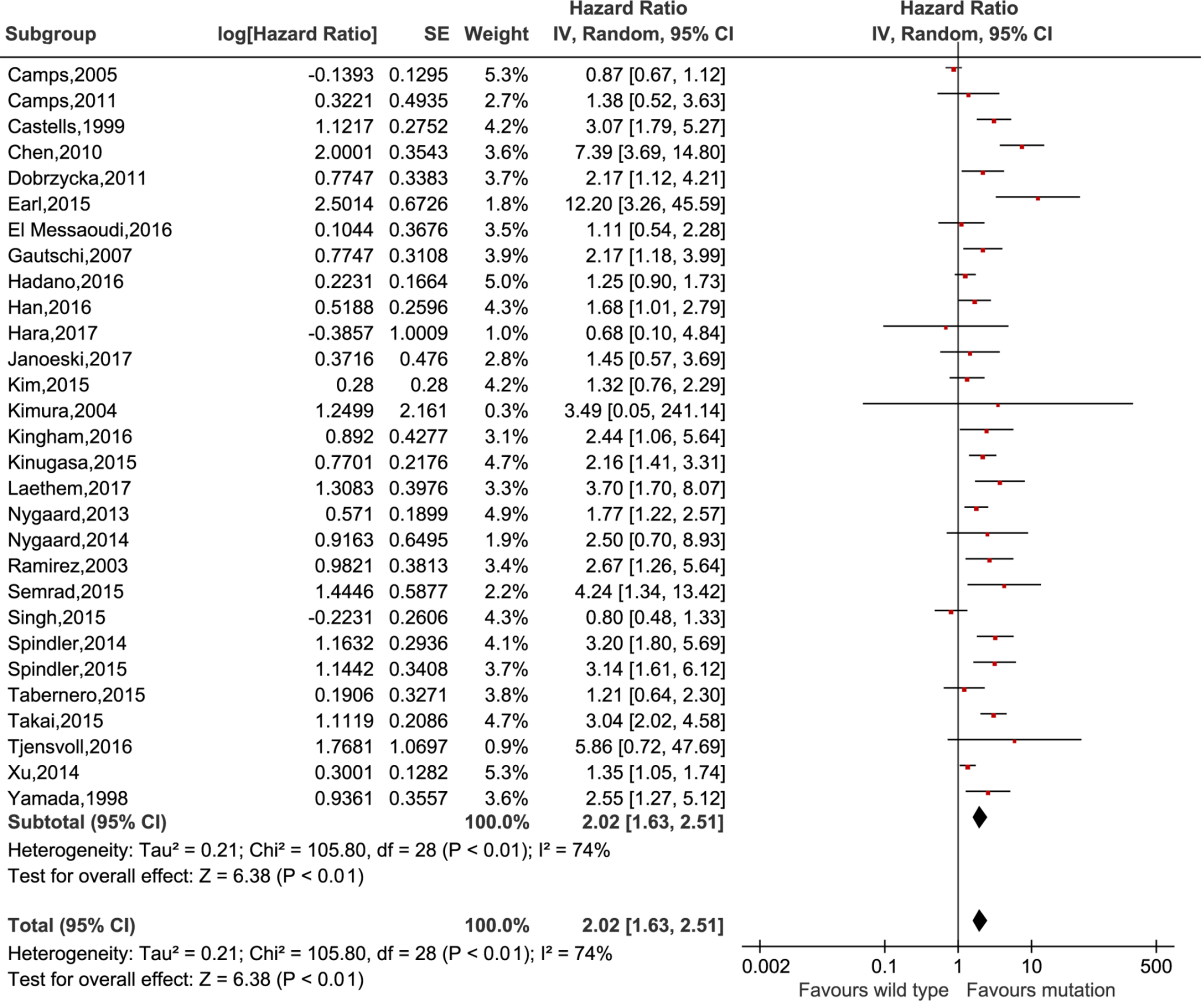
Forest plot for the association between *KRAS* mutation detected by cell-free DNA and overall survival in cancer patients.

**Fig 3:**
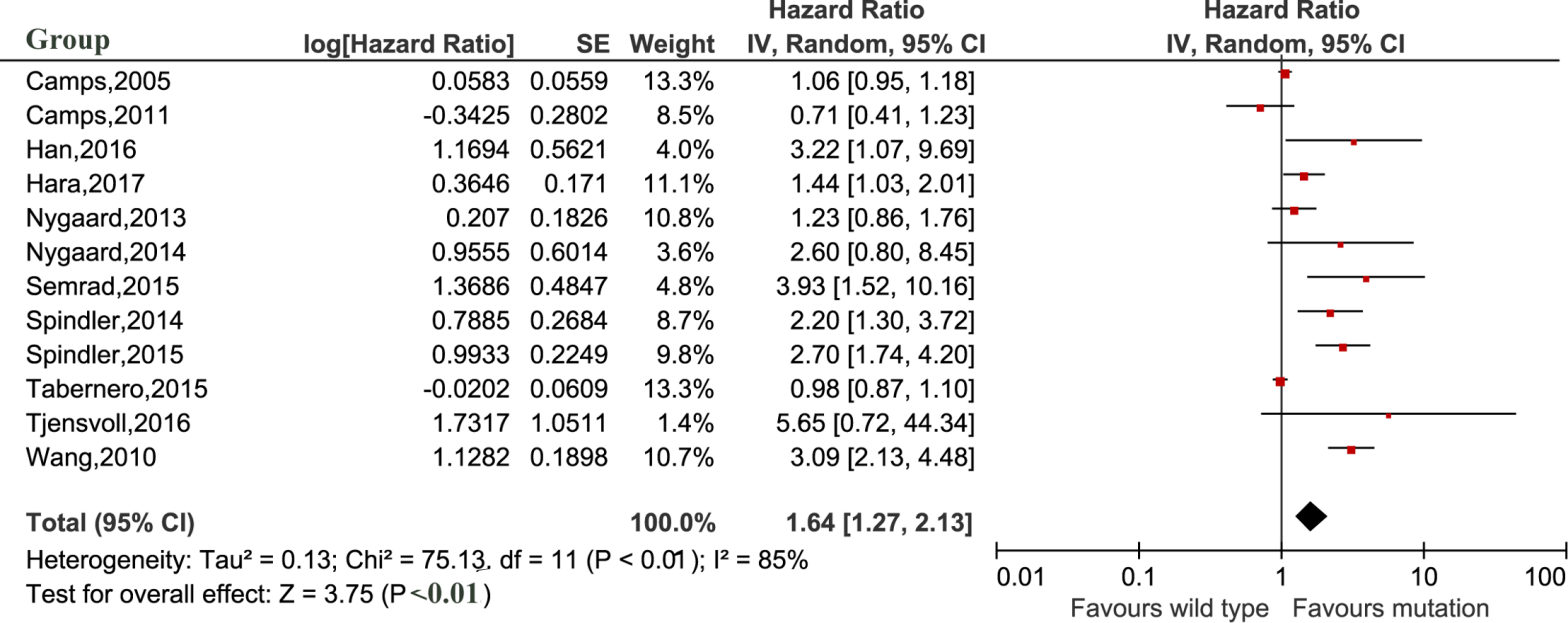
Forest plot for the association between *KRAS* mutation detected by cell-free DNA and progression free survival in cancer patients.

**Fig 4:**
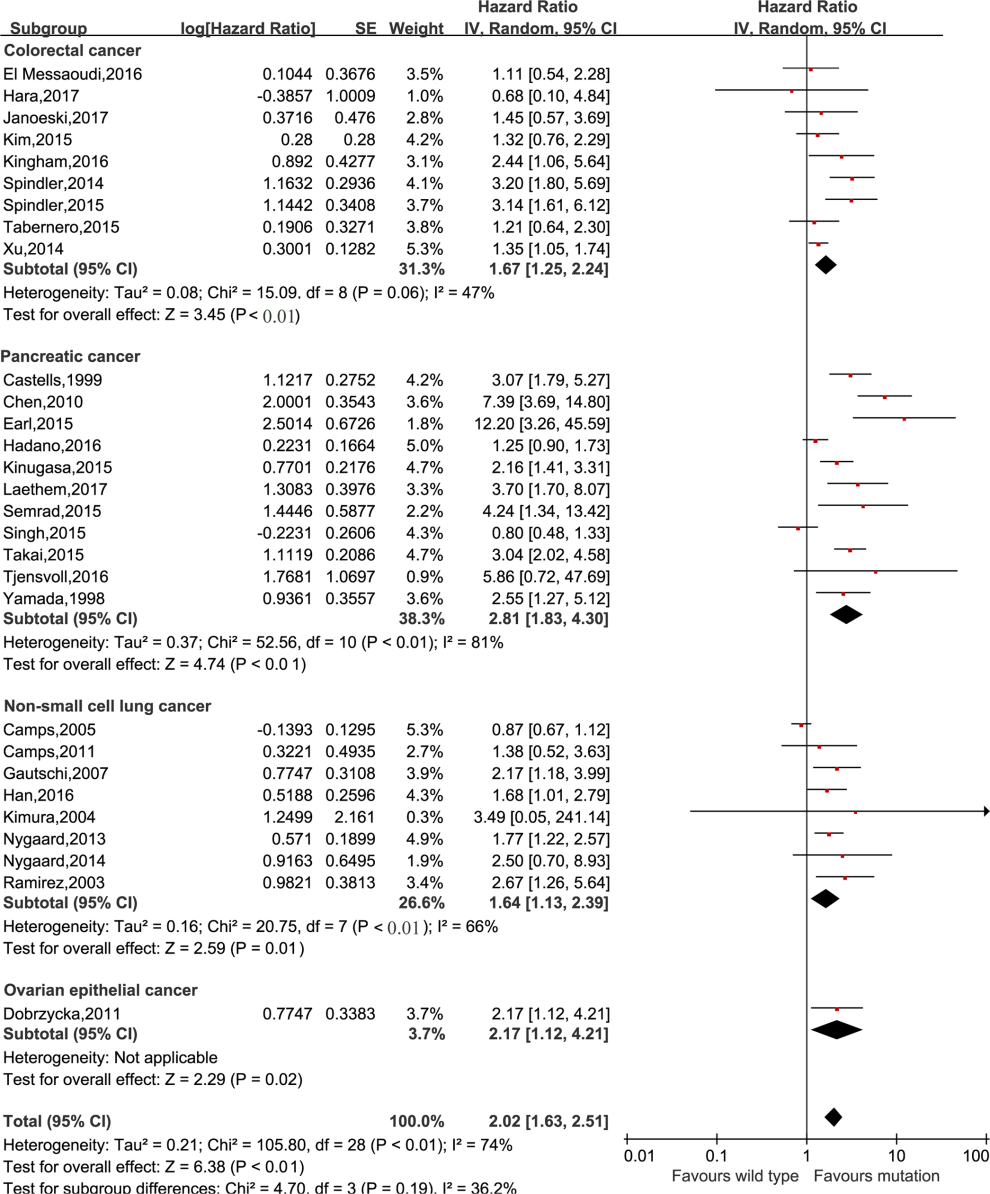
Forest plot for the subgroup analysis of cancer types.

**Fig 5:**
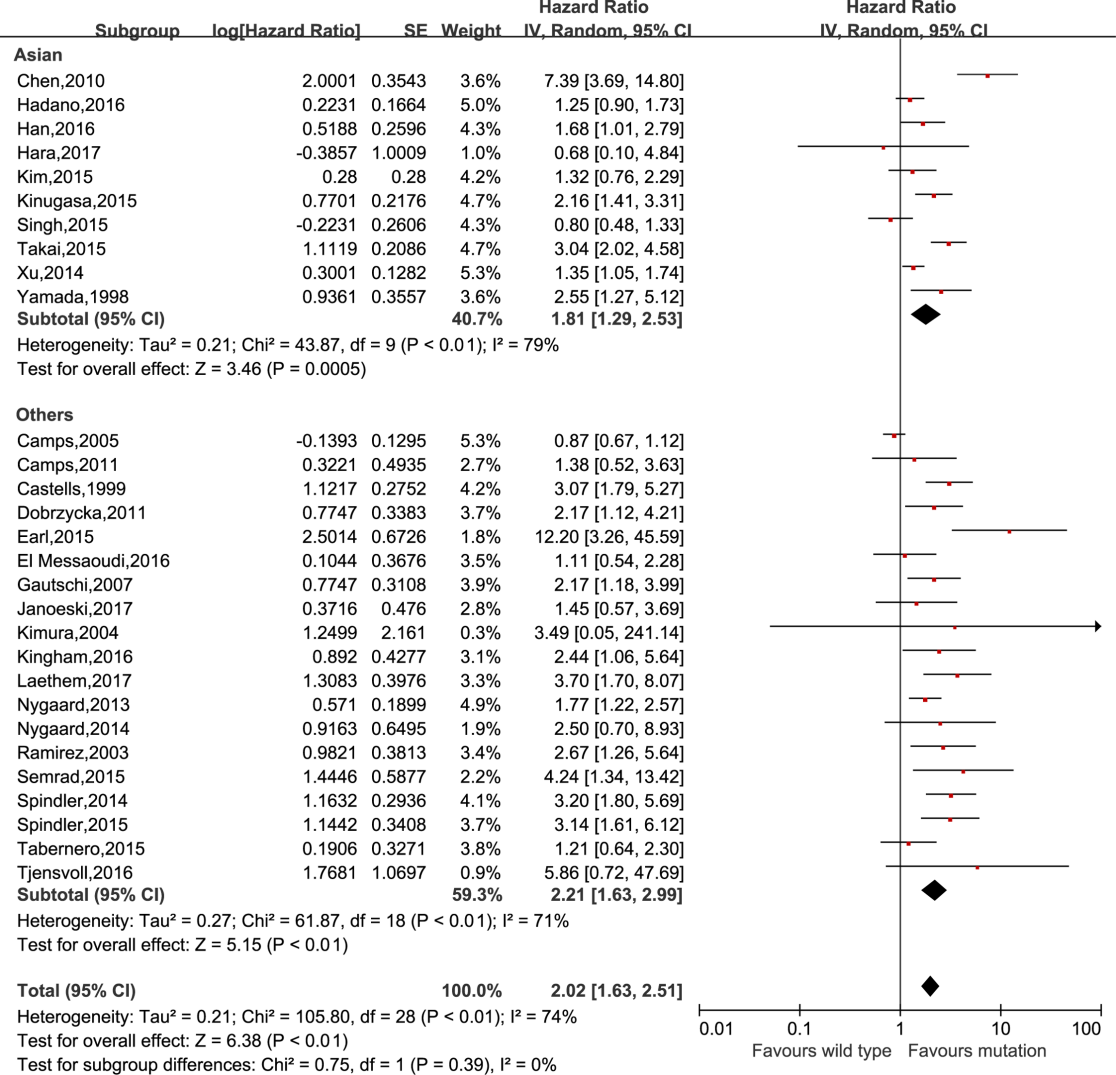
Forest plot for the subgroup analysis of ethnicity.

The meta-analysis was performed to investigate the prognostic value of *KRAS* mutation detected by cfDNA on survival in cancer patients. Our analysis showed that *KRAS* mutation detected by cfDNA was associated with a poorer survival in cancer patients for OS and PFS (HR = 2.02, 95% CI 1.63–2.51, *P*<0.01 and HR = 1.64, 95% CI 1.27–2.13, *P*<0.01, respectively) (Figs 2 and 3). In subgroup analyses, *KRAS* mutation detected by cfDNA in pancreatic cancer, colorectal cancer, non-small cell lung cancer and ovarian epithelial cancer had HRs of 2.81 (95% CI 1.83–4.30, *P*<0.01), 1.67 (95% CI 1.25–2.42, *P*<0.01), 1.64 (95% CI 1.13–2.39, *P* = 0.01) and 2.17 (95% 1.12–4.21, *p* = 0.02) (shown in Fig 4), respectively. Additionally, the ethnicity didn`t influence the prognostic value of *KRAS* mutation detected by cfDNA in cancer patients. *KRAS* mutation detected by cfDNA was a significant prognostic biomarker in cancer patients either in Asian (HR 1.81, 95% CI 1.29–2.53, *P*<0.01) or others population (HR 2.21, 95% CI 1.63–2.51, *P*<0.01) (Fig 5).

### Sensitivity analysis

Sensitivity analyses were presented in Table 2. Firstly, the sensitivity analysis was performed through removing one single study one by one from the overall pooled analysis. The results showed that there was no significant alteration of the pooled HRs after removing one single study in turn which indicating the results of our meta-analysis was relative stable (data not showed). Additionally, studies with reported HRs of OS tended to have higher HRs compared with studies with recomputed HRs using Parmar’s method (2.24 vs 1.76, *p* = 0.24) for all studies. There was no significant difference compared multivariate HRs with univariate HRs (2.53 vs 2.29, *p* = 0.74). For samples collection, no significant difference between serum samples and plasma samples was observed (HRs, 1.65 vs 2.13, *p* = 0.37).

**Table 2:** The sensitivity analysis for the meta-analysis.

Subgroup	HR (95%CI)	*p* value
**Type of publication**		
Reported	2.24 (1.66–3.01)	0.27
Recalculated (by Parmar’s method)	1.76 (1.30–2.40)	
**Analysis of hazard ratio**		
Multivariate	2.53 (1.66–3.86)	0.74
Univariate	2.29 (1.50–3.50)	
**Sample collection**		
Serum	1.65 (0.99–2.74)	0.37
Plasma	2.13 (1.69–2.70)	

### Publication bias

**Fig 6:**
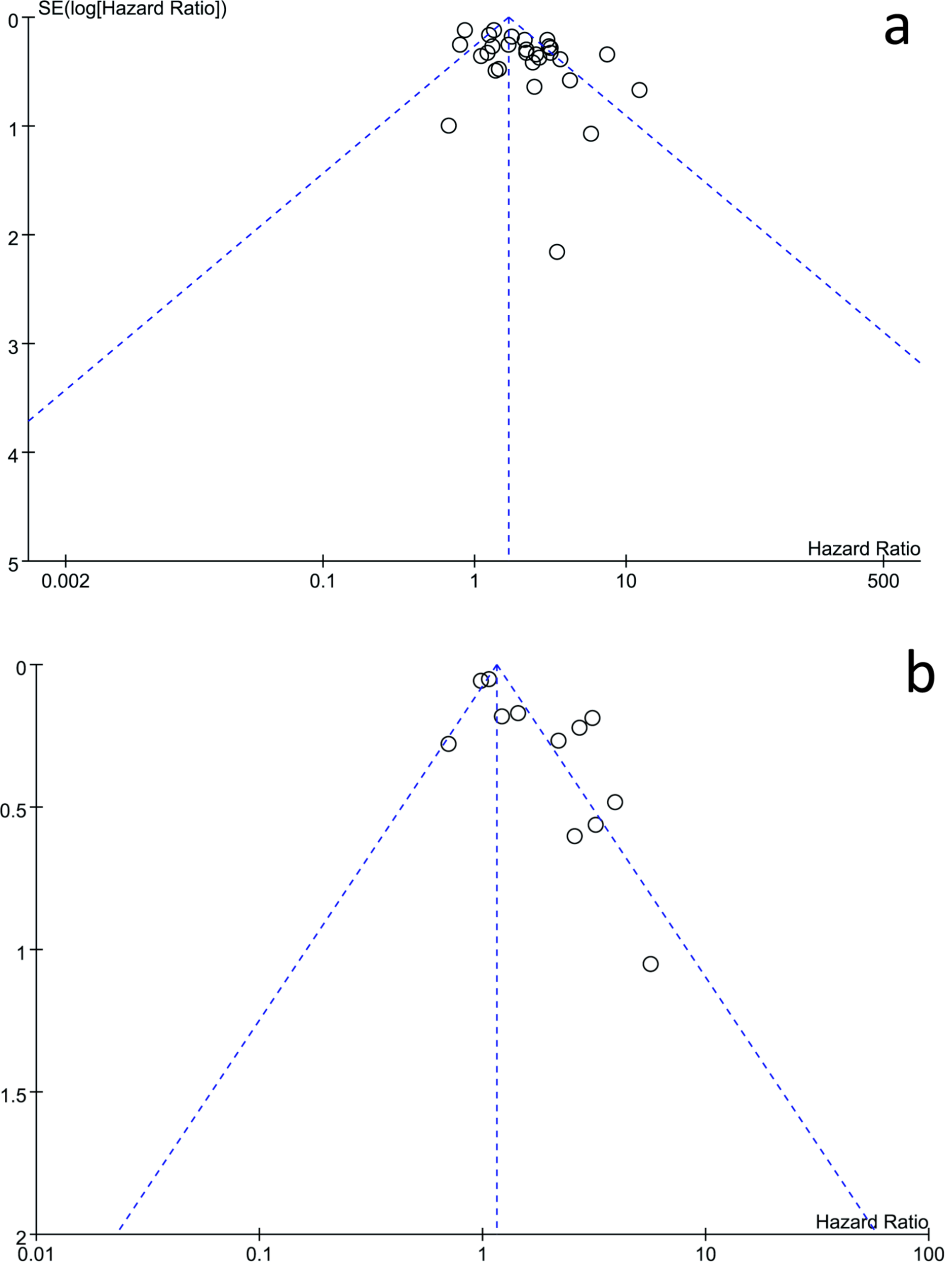
Funnel plot of the association between *KRAS* mutation detected by cell-free DNA and survival in cancer patients for publication bias. a, overall survival; b, progression-free survival.

The funnel plot and Egger’s test were used to assess the publication bias of the eligible studies. It seemed that the shape of the funnel plot was not symmetrical (shown in Fig 6A and 6B). In addition, the Egger’s test suggested that publication bias was existed (*P*<0.05).

## Discussion

Circulating cell-free DNA (cfDNA), which exists as small DNA fragments in blood, could be isolated from serum or plasma by less-invasive approach to diagnosis cancers, detect drug resistance and overcome the problem of tumor heterogeneity[44–46]. *KRAS* mutation is one of the most frequent molecular abnormalities found in several types of cancer such as pancreatic cancer, colorectal cancer, non-small cell lung cancer [47]. Spindler et al reported that there was strong relationship between the plasma levels of total cfDNA and the plasma *KRAS* mutated alleles in metastatic colorectal cancer [35]. Several studies found that cfDNA and the presence of mutant *KRAS* in plasma or serum cfDNA was significantly associated with the metastasis in patients with cancer [28,33,38,48]. In recent years, many studies found that *KRAS* mutation was associated with the recurrence [49–51] and with survival prognosis in various types of cancer [32,35][38], but several studies suggested that *KRAS* mutation in cfDNA was not associated with survival outcome of patients with pancreatic, lung or colon cancer [14,25,33]. The prognostic values of *KRAS* mutations in cfDNA as a biomarker remain to confirm. A meta-analysis had clarified that *KRAS* mutations in cfDNA had a more significant impact on overall survival of patients with pancreatic cancer compared with *KRAS* mutation detected in tumor tissue [7]. Our results indicated that *KRAS* detected in cfDNA was a prognostic marker for OS and PFS of pancreatic cancer, colorectal cancer and NSCLC. But another meta-analysis could not support *KRAS* mutation as survival marker in NSCLC [52]. One reason might be that more studies was included in our study (8 publications) compared with previous studies (4 publications). Maybe, large-scaled clinicltrials are necessary to confirm our results.

Currently, gene type analysis of tumor tissue is becoming a common practice in the clinical oncology, but there are some disadvantages such as tumor heterogeneity and samples being difficult to obtain. On the contrary, cfDNA is a non-invasive procedure and its samples would be easy to be collected [44,53]. Thus, considering the tumor heterogeneity of tumor tissue and the advantages of cfDNA, the cfDNA was selected according to the sample source in the present study. Furthermore, KRAS mutations in cfDNA is high correlated with mutations detected in the matched tumors [33,41]. Our meta-analysis showed that *KRAS* mutation detected in cfDNA was a significant prognostic biomarker of cancer patients, especially in pancreatic cancer.

Studies have suggested that the level of cfDNA is increased in both cancer patient and in various non-malignant pathological conditions compared to healthy individuals [48]. Even minority of healthy subjects demonstrated mutant KRAS in cfDNA [54], so the *KRAS* mutation used for disease diagnosis should be cautious. Generally, the prevalence of *KRAS* mutations in tumor tissues was high than that of cfDNA in pancreatic cancer and colorectal cancer [7,42]. Previous studies have suggested that the detection of tumor derived cfDNA is more trend in the setting of large tumor burden and tumor high turnover which are both independent predictors of a poor prognosis [38,48,55]. Result from Spindler et al [35] indicated there was strong relationship between the plasma levels of total cfDNA and the plasma *KRAS* mutated alleles in metastatic colorectal cancer. Several studies found that cfDNA and the presence of mutant *KRAS* in plasma or serum cfDNA was significantly associated with the metastasis in patients with cancer [28,33,38,48]. However, others reported there were no association observed between *KRAS* mutation and age, sex, tumor stage, histopathologic type and so on in advanced cancers [25,41]. In order to clarify this issue, we conducted a sensitivity analysis to compare univariate and multivariate analysis about prognostic value of *KRAS* in cfDNA in sensitivity analysis, and results proved *KRAS* mutation in cfDNA was an independent marker of poor prognosis of overall survival (HR = 2.53, 95%CI: 1.66–3.86, *p*<0.01).

There were some limitations in the present meta-analysis. At first, most of the studies included in our meta-analysis were retrospective, which may bring about some potential bias. Second, some studies of other databases might be lost and some relevant studies were excluded in our meta-analysis because of the publication limitations or incompletely raw data. Third, several studies didn’t report HR and its related 95% CI and needed to be calculated according to Parmar’s method [9] which might cause imprecise values and potential bias. In addition, there was heterogeneity existed in the eligible studies which might lead to an inaccurate conclusion.

In conclusion, our meta-analysis demonstrated that *KRAS* mutation detected in cfDNA was a prognostic biomarker in cancer patients. Its prognostic value was different in different types of cancer. However, because of the limitations existed in our meta-analysis, more studies are still needed to support our conclusions.

## Supporting information

S1 Table The search strategy of the prognostic value of *KRAS* mutation detected by cell-free DNA in cancer patients. (PDF)

S2 Table Application of the quality assessment tool NOS to the studies included in the meta-analysis. (PDF)

S3 Table PRISMA 2009 checklist. (DOC)

## References

[1] Benson AB, 3rd, Venook AP, Cederquist L, Chan E, Chen YJ, Cooper HS, et al Colon Cancer, Version 1.2017, NCCN Clinical Practice Guidelines in Oncology. J Natl Compr Canc Netw. 2017; 15: 370–398. 28275037 10.6004/jnccn.2017.0036

[2] Zeitouni D, Pylayeva-Gupta Y, Der CJ, Bryant KL. KRAS Mutant Pancreatic Cancer: No Lone Path to an Effective Treatment. Cancers (Basel). 2016; 8: E45 doi: 10.3390/cancers8040045 27096871 10.3390/cancers8040045 PMC4846854

[3] Zhou B, Der CJ, Cox AD. The role of wild type RAS isoforms in cancer. Semin Cell Dev Biol. 2016; 58: 60–69. doi: 10.1016/j.semcdb.2016.07.012 27422332 10.1016/j.semcdb.2016.07.012 PMC5028303

[4] Qian J, Niu J, Li M, Chiao PJ, Tsao MS. In vitro modeling of human pancreatic duct epithelial cell transformation defines gene expression changes induced by K-ras oncogenic activation in pancreatic carcinogenesis. Cancer Res. 2005; 65: 5045–5053. doi: 10.1158/0008-5472.CAN-04-3208 15958547 10.1158/0008-5472.CAN-04-3208

[5] Brembeck FH, Schreiber FS, Deramaudt TB, Craig L, Rhoades B, Swain G, et al The mutant K-ras oncogene causes pancreatic periductal lymphocytic infiltration and gastric mucous neck cell hyperplasia in transgenic mice. Cancer Res. 2003; 63: 2005–2009. 12727809

[6] Collins MA, Bednar F, Zhang Y, Brisset JC, Galban S, Galban CJ, et al Oncogenic Kras is required for both the initiation and maintenance of pancreatic cancer in mice. J Clin Invest. 2012; 122: 639–653. doi: 10.1172/JCI59227 22232209 10.1172/JCI59227 PMC3266788

[7] Li T, Zheng Y, Sun H, Zhuang R, Liu J, Liu T, et al K-Ras mutation detection in liquid biopsy and tumor tissue as prognostic biomarker in patients with pancreatic cancer: a systematic review with meta-analysis. Med Oncol. 2016; 33: 61 doi: 10.1007/s12032-016-0777-1 27225938 10.1007/s12032-016-0777-1

[8] Stang A. Critical evaluation of the Newcastle-Ottawa scale for the assessment of the quality of nonrandomized studies in meta-analyses. Eur J Epidemiol. 2010; 25: 603–605. doi: 10.1007/s10654-010-9491-z 20652370 10.1007/s10654-010-9491-z

[9] Parmar MK, Torri V, Stewart L. Extracting summary statistics to perform meta-analyses of the published literature for survival endpoints. Stat Med. 1998; 17: 2815–2834. 9921604 10.1002/(sici)1097-0258(19981230)17:24<2815::aid-sim110>3.0.co;2-8

[10] Tierney JF, Stewart LA, Ghersi D, Burdett S, Sydes MR. Practical methods for incorporating summary time-to-event data into meta-analysis. Trials. 2007; 8: 16 doi: 10.1186/1745-6215-8-16 17555582 10.1186/1745-6215-8-16 PMC1920534

[11] Begg CB, Mazumdar M. Operating characteristics of a rank correlation test for publication bias. Biometrics. 1994; 50: 1088–1101. 7786990

[12] Egger M, Davey Smith G, Schneider M, Minder C. Bias in meta-analysis detected by a simple, graphical test. Bmj. 1997; 315: 629–634. 9310563 10.1136/bmj.315.7109.629 PMC2127453

[13] Camps C, Jantus-Lewintre E, Cabrera A, Blasco A, Sanmartin E, Gallach S, et al The identification of KRAS mutations at codon 12 in plasma DNA is not a prognostic factor in advanced non-small cell lung cancer patients. Lung Cancer. 2011; 72: 365–369. doi: 10.1016/j.lungcan.2010.09.005 21074889 10.1016/j.lungcan.2010.09.005

[14] Camps C, Sirera R, Bremnes R, Blasco A, Sancho E, Bayo P, et al Is there a prognostic role of K-ras point mutations in the serum of patients with advanced non-small cell lung cancer? Lung Cancer. 2005; 50: 339–346. doi: 10.1016/j.lungcan.2005.06.007 16139926 10.1016/j.lungcan.2005.06.007

[15] Castells A, Puig P, Mora J, Boadas J, Boix L, Urgell E, et al K-ras mutations in DNA extracted from the plasma of patients with pancreatic carcinoma: diagnostic utility and prognostic significance. J Clin Oncol. 1999; 17: 578–584. doi: 10.1200/JCO.1999.17.2.578 10080602 10.1200/JCO.1999.17.2.578

[16] Chen H, Tu H, Meng ZQ, Chen Z, Wang P, Liu LM. K-ras mutational status predicts poor prognosis in unresectable pancreatic cancer. Eur J Surg Oncol.2010; 36: 657–662. doi: 10.1016/j.ejso.2010.05.014 20542658 10.1016/j.ejso.2010.05.014

[17] Dobrzycka B, Terlikowski SJ, Kinalski M, Kowalczuk O, Niklinska W, Chyczewski L. Circulating free DNA and p53 antibodies in plasma of patients with ovarian epithelial cancers. Ann Oncol 2011; 22: 1133–1140. doi: 10.1093/annonc/mdq584 21098618 10.1093/annonc/mdq584

[18] Earl J, Garcia-Nieto S, Martinez-Avila JC, Montans J, Sanjuanbenito A, Rodriguez-Garrote M, et al Circulating tumor cells (Ctc) and kras mutant circulating free Dna (cfdna) detection in peripheral blood as biomarkers in patients diagnosed with exocrine pancreatic cancer.BMC Cancer. 2015; 15: 797 doi: 10.1186/s12885-015-1779-7 26498594 10.1186/s12885-015-1779-7 PMC4619983

[19] El Messaoudi S, Mouliere F, Du Manoir S, Bascoul-Mollevi C, Gillet B, Nouaille M, et al Circulating DNA as a Strong Multimarker Prognostic Tool for Metastatic Colorectal Cancer Patient Management Care. Clin Cancer Res. 2016; 22: 3067–3077. doi: 10.1158/1078-0432.CCR-15-0297 26847055 10.1158/1078-0432.CCR-15-0297

[20] Gautschi O, Huegli B, Ziegler A, Gugger M, Heighway J, Ratschiller D, et al Origin and prognostic value of circulating KRAS mutations in lung cancer patients. Cancer Letter 2007; 254: 265–273. 10.1016/j.canlet.2007.03.008 17449174

[21] Hadano N, Murakami Y, Uemura K, Hashimoto Y, Kondo N, Nakagawa N, et al Prognostic value of circulating tumour DNA in patients undergoing curative resection for pancreatic cancer. Br J Cancer. 2016; 115: 59–65. doi: 10.1038/bjc.2016.175 27280632 10.1038/bjc.2016.175 PMC4931379

[22] Han JY, Choi JJ, Kim JY, Han YL, Lee GK. PNA clamping-assisted fluorescence melting curve analysis for detecting EGFR and KRAS mutations in the circulating tumor DNA of patients with advanced non-small cell lung cancer. BMC Cancer. 2016; 16: 627 doi: 10.1186/s12885-016-2678-2 27519791 10.1186/s12885-016-2678-2 PMC4983013

[23] Hara M, Nagasaki T, Shiga K, Takahashi H, Takeyama H. High serum levels of interleukin-6 in patients with advanced or metastatic colorectal cancer: the effect on the outcome and the response to chemotherapy plus bevacizumab. Surg Today. 2017; 47: 483–489. doi: 10.1007/s00595-016-1404-7 27549777 10.1007/s00595-016-1404-7

[24] Janowski E, Timofeeva O, Chasovskikh S, Goldberg M, Kim A, Banovac F, et al Yttrium-90 radioembolization for colorectal cancer liver metastases in KRAS wild-type and mutant patients: Clinical and ccfDNA studies. Oncol Rep. 2017; 37: 57–65. doi: 10.3892/or.2016.5284 28004119 10.3892/or.2016.5284 PMC5355723

[25] Kim ST, Chang WJ, Jin L, Sung JS, Choi YJ, Kim YH. Can serum be used for analyzing the KRAS mutation status in patients with advanced colorectal cancer?. Cancer Res Treat. 2015; 47: 796–803. doi: 10.4143/crt.2014.106 25687873 10.4143/crt.2014.106 PMC4614179

[26] Kimura T, Holland WS, Kawaguchi T, Williamson SK, Chansky K, Crowley JJ, et al Mutant DNA in plasma of lung cancer patients: potential for monitoring response to therapy. Ann N Y Acad Sci. 2004; 1022: 55–60. doi: 10.1196/annals.1318.010 15251940 10.1196/annals.1318.010

[27] Kinugasa H, Nouso K, Miyahara K, Morimoto Y, Dohi C, Tsutsumi K, et al Detection of K-ras gene mutation by liquid biopsy in patients with pancreatic cancer. Cancer. 2015; 121: 2271–2280. doi: 10.1002/cncr.29364 25823825 10.1002/cncr.29364

[28] Nygaard AD, Garm Spindler KL, Pallisgaard N, Andersen RF, Jakobsen A. The prognostic value of KRAS mutated plasma DNA in advanced non-small cell lung cancer.Oncol Rep. 2013; 31: 312–317. 10.1016/j.lungcan.2012.11.016 23238036

[29] Nygaard AD, Spindler KLG, Pallisgaard N, Andersen RF, Jakobsen A. Levels of cell-free DNA and plasma KRAS during treatment of advanced NSCLC. Oncol Rep. 2014; 31: 969–974. doi: 10.3892/or.2013.2906 24316734 10.3892/or.2013.2906

[30] Kingham TP, Nguyen HC, Zheng J, Konstantinidis IT, Sadot E, Shia J, et al MicroRNA-203 predicts human survival after resection of colorectal liver metastasis. Oncotarget. 2016; 8: 18821–18831. 10.18632/oncotarget.13816 PMC5386650 27935861

[31] Ramirez JL, Sarries C, de Castro PL, Roig B, Queralt C, Escuin D, et al Methylation patterns and K-ras mutations in tumor and paired serum of resected non-small-cell lung cancer patients. Cancer Lett. 2003; 193: 207–216. 12706879 10.1016/s0304-3835(02)00740-1

[32] Semrad T, Barzi A, Lenz HJ, Hutchins IM, Kim EJ, Gong IY, et al Pharmacodynamic separation of gemcitabine and erlotinib in locally advanced or metastatic pancreatic cancer: therapeutic and biomarker results. Int J Clin Oncol. 2015; 20: 518–524. doi: 10.1007/s10147-014-0730-2 25091263 10.1007/s10147-014-0730-2 PMC4318776

[33] Singh N, Gupta S, Pandey RM, Chauhan SS, Saraya A. High levels of cell-free circulating nucleic acids in pancreatic cancer are associated with vascular encasement, metastasis and poor survival. Cancer Invest. 2015; 33: 78–85. doi: 10.3109/07357907.2014.1001894 25647443 10.3109/07357907.2014.1001894

[34] Spindler KG, Appelt AL, Pallisgaard N, Andersen RF, Jakobsen A. KRAS-mutated plasma DNA as predictor of outcome from irinotecan monotherapy in metastatic colorectal cancer. Br J **Cancer**. 2013; 111: 3067–3072. 10.1038/bjc.2013.633 PMC3859936 24263065

[35] Spindler KL, Pallisgaard N, Appelt AL, Andersen RF, Schou JV, Nielsen D, et al Clinical utility of KRAS status in circulating plasma DNA compared to archival tumour tissue from patients with metastatic colorectal cancer treated with anti-epidermal growth factor receptor therapy. Eur J Cancer.2015; 51: 2678–2685. doi: 10.1016/j.ejca.2015.06.118 26508156 10.1016/j.ejca.2015.06.118

[36] Spindler KL, Pallisgaard N, Vogelius I, Jakobsen A. Quantitative cell-free DNA, KRAS, and BRAF mutations in plasma from patients with metastatic colorectal cancer during treatment with cetuximab and irinotecan. Clin Cancer Res. 2012; 18: 1177–1185. doi: 10.1158/1078-0432.CCR-11-0564 22228631 10.1158/1078-0432.CCR-11-0564

[37] Tabernero J, Lenz HJ, Siena S, Sobrero A, Falcone A, Ychou M, et al Analysis of circulating DNA and protein biomarkers to predict the clinical activity of regorafenib and assess prognosis in patients with metastatic colorectal cancer: A retrospective, exploratory analysis of the CORRECT trial. Lancet Oncol. 2015; 16: 937–948. doi: 10.1016/S1470-2045(15)00138-2 26184520 10.1016/S1470-2045(15)00138-2 PMC7513622

[38] Takai E, Totoki Y, Nakamura H, Morizane C, Nara S, Hama N, et al Clinical utility of circulating tumor DNA for molecular assessment in pancreatic cancer. Sci Rep. 2015; 5: 18425 doi: 10.1038/srep18425 26669280 10.1038/srep18425 PMC4680882

[39] Tjensvoll K, Lapin M, Buhl T, Oltedal S, Berry KS-O, Gilje B, et al Clinical relevance of circulating KRAS mutated DNA in plasma from patients with advanced pancreatic cancer. Molecular Oncology. 2016; 10: 635–643. doi: 10.1016/j.molonc.2015.11.012 26725968 10.1016/j.molonc.2015.11.012 PMC5423145

[40] Van Laethem JL, Riess H, Jassem J, Haas M, Martens UM, Weekes C, et al Phase I/II Study of Refametinib (BAY 86–9766) in Combination with Gemcitabine in Advanced Pancreatic cancer. Target Oncol. 2017; 12: 97–109. doi: 10.1007/s11523-016-0469-y 27975152 10.1007/s11523-016-0469-y

[41] Wang S, An T, Wang J, Zhao J, Wang Z, Zhuo M, et al Potential clinical significance of a plasma-based KRAS mutation analysis in patients with advanced non-small cell lung cancer. Clin Cancer Res. 2010; 16: 1324–1330. doi: 10.1158/1078-0432.CCR-09-2672 20145159 10.1158/1078-0432.CCR-09-2672

[42] Xu JM, Liu XJ, Ge FJ, Lin L, Wang Y, Sharma MR, et al KRAS mutations in tumor tissue and plasma by different assays predict survival of patients with metastatic colorectal cancer.J Exp Clin Cancer Res. 2014; 33: 104 doi: 10.1186/s13046-014-0104-7 25491325 10.1186/s13046-014-0104-7 PMC4272803

[43] Yamada T, Nakamori S, Ohzato H, Oshima S, Aoki T, Higaki N, et al Detection of K-ras gene mutations in plasma DNA of patients with pancreatic adenocarcinoma: correlation with clinicopathological features. Clin Cancer Res. 1998; 4: 1527–1532. 9626473

[44] Diaz LA Jr., Bardelli A. Liquid biopsies: genotyping circulating tumor DNA. J Clin Oncol. 2014; 32: 579–586. doi: 10.1200/JCO.2012.45.2011 24449238 10.1200/JCO.2012.45.2011 PMC4820760

[45] Riediger AL, Dietz S, Schirmer U, Meister M, Heinzmann-Groth I, Schneider M, et al Mutation analysis of circulating plasma DNA to determine response to EGFR tyrosine kinase inhibitor therapy of lung adenocarcinoma patients. Sci Rep. 2016; 6: 33505 doi: 10.1038/srep33505 27640882 10.1038/srep33505 PMC5027592

[46] Oxnard GR, Paweletz CP, Kuang Y, Mach SL, O'Connell A, Messineo MM, et al Noninvasive Detection of Response and Resistance in EGFR-Mutant Lung Cancer Using Quantitative Next-Generation Genotyping of Cell-Free Plasma DNA. Clinical Cancer Research. 2014; 20: 1698–1705. doi: 10.1158/1078-0432.CCR-13-2482 24429876 10.1158/1078-0432.CCR-13-2482 PMC3959249

[47] Prior IA, Lewis PD, Mattos C A comprehensive survey of Ras mutations in cancer. Cancer Res. 2012; 72: 2457–2467. doi: 10.1158/0008-5472.CAN-11-2612 22589270 10.1158/0008-5472.CAN-11-2612 PMC3354961

[48] Spindler KL, Appelt AL, Pallisgaard N, Andersen RF, Brandslund I, Jakobsen A, et al Cell-free DNA in healthy individuals, noncancerous disease and strong prognostic value in colorectal cancer. Int J Cancer. 2014; 135: 2984–2991. doi: 10.1002/ijc.28946 24798213 10.1002/ijc.28946

[49] Wang JSZ, Sausen M, Parpart-Li S, Murphy DM, Velculescu VE, Wood LD, et al. Circulating tumor DNA (ctDNA) as a prognostic marker for recurrence in resected pancreas cancer. 2015 Annual Meeting of the American Society of Clinical Oncology, ASCO Chicago, IL United States. 15 SUPPL. 1.

[50] Garcia-Murillas I, Schiavon G, Weigelt B, Ng C, Hrebien S, Cutts RJ, et al Mutation tracking in circulating tumor DNA predicts relapse in early breast cancer. Sci Transl Med. 2015; 7: 302ra133 doi: 10.1126/scitranslmed.aab0021 26311728 10.1126/scitranslmed.aab0021

[51] Lin SH, Xu T, He J, Banks K, Lanman RB, Sebisanovic D, et al. Dynamic changes in cell-free circulating tumor dna to track tumor response and risk of recurrence in stage III non-small cell lung cancer. 16th World Conference on Lung Cancer Denver, CO United States. 9 SUPPL. 2015; pp. S287.

[52] Ai B, Liu H, Huang Y, Peng P. Circulating cell-free DNA as a prognostic and predictive biomarker in non-small cell lung cancer. Oncotarget. 2016; 7: 44583–44595. doi: 10.18632/oncotarget.10069 27323821 10.18632/oncotarget.10069 PMC5190120

[53] Hofman P, Popper HH. Pathologists and liquid biopsies: to be or not to be? Virchows Arch. 2016; 469: 601–609. doi: 10.1007/s00428-016-2004-z 27553354 10.1007/s00428-016-2004-z

[54] Yang S, Che SP, Kurywchak P, Tavormina JL, Gansmo LB, Correa de Sampaio P, et al Detection of mutant KRAS and TP53 DNA in circulating exosomes from healthy individuals and patients with pancreatic cancer. Cancer Biol Ther. 2017; 18: 158–165. doi: 10.1080/15384047.2017.1281499 28121262 10.1080/15384047.2017.1281499 PMC5389423

[55] Pereira E, Camacho-Vanegas O, Anand S, Sebra R, Catalina Camacho S, Garnar-Wortzel L, et al Personalized Circulating Tumor DNA Biomarkers Dynamically Predict Treatment Response and Survival In Gynecologic Cancers. PLoS One. 2015;10: e0145754 doi: 10.1371/journal.pone.0145754 26717006 10.1371/journal.pone.0145754 PMC4696808

