# Supplementary material for: The prognostic value of KRAS mutation by cell-free DNA in cancer patients: A systematic review and meta-analysis
Source: PLoS One. 2017 Aug 10;12(8):e0182562. doi: 10.1371/journal.pone.0182562 (PMC5552123; doi:10.1371/journal.pone.0182562)
Supplement: S1 Table — (PDF) [file pone.0182562.s001.pdf]

S1 Table. The search strategy of the prognostic value of K-Ras mutation detected by cell-free DNA in cancer patients

EMBASE Ovid (to 2016 June 20)

1. Exp neoplasms/
2. (malignan$ or neoplasm$ or cancer$ or carcinoma$ or adenocarcinoma$ or tumour$ or tumor$ or leukemia$ myeloma$ or lymphoma$ or melanoma$ or sarcoma$ or blastoma$ or fibrous histiotoma$ or neurofibrosarcoma$ or neurilemmoma$ or glioma$ or medulloblastoma$ or meningioma$ or neuroblastoma$ or chorio-epithelioma$ ormalignant mole$ ormalignant granular cell tumor$ or seminoma$ or dysgerminoma$ or teratoma$).tw
3. 1 or 2
4. K-ras.mp
5. KRAS.mp
6. kirsten-ras.mp
7. Kirsten ras.mp
8. ki-ras.mp
9. 4 or 5 or 6 or 7 or 8
10. liquid biopsy.mp
11. liquid biopsies.mp

12. serum.mp<br>13. plasma.mp<br>14. cell free DNA.mp<br>15. cell-free DNA.mp<br>16. cfDNA.mp<br>17. cell-free plasma DNA.mp<br>18. 10 or 11 or 12 or 13 or 14 or 15 or 16 or 17<br>19. exp prognosis/<br>20. exp morbidity/<br>21. exp mortality/<br>22. exp recurrence/<br>23. exp metastasis/<br>24. exp survival analysis/<br>25. prognos$.tw<br>26. predict$.tw<br>27. death$.tw<br>28. surviv$.tw<br>29. 19 or 20 or 21 or 22 or 23 or 24 or 25 or 26 or 27 or 28<br>30. 3 and 9 and 18 and 29
**PubMed (to 2017 March)**<br>1. "Neoplasms"[Mesh] OR malignant OR malignancy OR neoplasm? OR cancer? OR carcinoma? OR adenocarcinoma? OR tumour? OR tumor? OR leukemia? OR

myeloma? OR hepatoma? OR lymphoma? OR melanoma? OR sarcoma? OR blastoma? OR fibrous histiocytoma? OR neurofibrosarcoma? OR neurilemmoma? OR glioma? OR glioblastoma? OR medulloblastoma? OR meningioma? OR neuroblastoma? OR chorio-epithelioma? OR malignant mole? OR malignant granular cell tumor? OR seminoma? OR dysgerminoma? OR teratoma?

2. K-ras OR KRAS OR kirsten-ras OR Kirsten ras OR ki-ras
3. "liquid biopsy" OR "liquid biopsies" OR serum OR plasma OR "cell free DNA" OR "cell-free plasma DNA" OR "cell-free DNA" OR cfDNA
4. prognosis OR prognostic OR predict OR predictive OR prediction OR morbidity OR mortality OR death OR recurrence OR recurrent OR metastasis OR metastatic OR survival OR survive OR "survival analysis"
5. 1 and 2 and 3 and 4

Web of Science (to 2016 June 20)

1.(neoplasms or malignan or neoplasm or cancer or carcinoma or adenocarcinoma or tumour or tumor or leukemia myeloma or lymphoma or melanoma or sarcoma or blastoma or fibrous histiotoma or neurofibrosarcoma or neurilemmoma or glioma or medulloblastoma or meningioma or neuroblastoma or chorio-epithelioma ormalignant mole ormalignant granular cell tumor or seminoma or dysgerminoma or teratoma)

2.K- ras OR KRAS OR kirsten-ras OR Kirsten ras OR ki-ras

3. "liquid biopsy" OR "liquid biopsies" OR serum OR plasma OR "cell free DNA" OR "cell-free plasma DNA" OR "cell-free DNA" OR cfDNA

4. (prognosis OR prognostic OR predict OR predictive OR prediction OR morbidity

OR mortality OR death OR recurrence OR recurrent OR metastasis OR metastatic OR survival OR survive OR survival analysis)<br>5. 1 and 2 and 3 and 4
**<u>Cochrane library (to 2016 June 20)</u>**<br>#1 neoplasm*<br>#2 malignan or neoplasm or cancer or carcinoma or adenocarcinoma or tumour or tumor or leukemia myeloma or lymphoma or melanoma or sarcoma or blastoma or fibrous histiotoma or neurofibrosarcoma or neurilemmoma or glioma or medulloblastoma or meningioma or neuroblastoma or chorio-epithelioma ormalignant mole ormalignant granular cell tumor or seminoma or dysgerminoma or teratoma<br>#3 (#1 or #2)<br>#4 K- ras<br>#5 KRAS<br>#6 kirsten-ras<br>#7 Kirsten ras<br>#8 ki-ras<br>#9 (#4 or #5 or #6 or #7 or #8)<br>#10 liquid biopsy<br>#11 liquid biopsies<br>#12 serum<br>#13 plasma<br>#14 cell free DNA

#15 cell-free plasma DNA

#16 cell-free DNA

#17 cfDNA

#18 (#10 or #11 or #12 or #13 or #14 or #15 or #16 or #17)

#19 prognosis

#20 morbidity

#21 mortality

#22 recurrence

#23 metastasis

#24 survival analysis

#25 prognos*

#26 predict*

#27 death*

#28 surviv*

#29 (#19 or #20 or #21 or #22 or #23 or #24 or #25 or #26 or #27 or #28)

#30 (#3 and #9 and #18 and #29)
