# Supplementary material for: The prognostic value of KRAS mutation by cell-free DNA in cancer patients: A systematic review and meta-analysis
Source: PLoS One. 2017 Aug 10;12(8):e0182562. doi: 10.1371/journal.pone.0182562 (PMC5552123; doi:10.1371/journal.pone.0182562)
Supplement: S2 Table — (PDF) [file pone.0182562.s002.pdf]

S2 Table. Application of the quality assessment tool NOS to the studies included in the meta-analysis

Items	Subgroups	Details	No 1	No 2	No 3	No 4	No 5	No 6	No 7	No 8	No 9	No 10	No 11	No 12	No 13	No 14	No 15	No 16	No 17	No 18	No 19	No 20	No 21	No 22	No 23	No 24	No 25	No 26	No 27	No 28	No 29	No 30
			Camps, 2005	Camps, 2011	Castells, 1999	Chen, 2010	Dobrzycka, 2011	Earl, 2015	El Messaoudi, 2016	Gautschi, 2007	Hadano, 2016	Han,2016	Hara,2017	Janowski, 2017	Kim,2015	Kimura, 2004	Kingham,2016	Kinugasa, 2015	Laethem,2017	Nygaard, 2013	Nygaard, 2014	Ramirez, 2003	Semrad, 2015	Singh, 2015	Spindler, 2014	Spindler, 2015	Tabernero, 2015	Takai, 2015	Tjensvoll, 2016	Wang, 2010	Xu, 2014	Yamada, 1998
Selection	1) Representativeness of the exposed cohort	a) truly representative of the average _______ (describe) in the community *	*	*	*	*	*	*	*	*	*	*		*	*	*	*	*	*	*	*	*	*	*	*	*	*	*	*	*	*	*
		b) somewhat representative of the average _______in the community*																														
		c) selected group of users eg nurses, volunteers																														
		d) no description of the derivation of the cohort																														
	2) Selection of the non exposed cohort	a) drawn from the same community as the exposed cohort *	*	*	*	*	*	*	*	*	*	*	*	*	*	*	*	*	*	*	*	*	*	*	*	*	*	*	*	*	*	*
		b) drawn from a different source																														
		c) no description of the derivation of the non exposed cohort																														
	3) Ascertainment of exposure	a) secure record (eg surgical records) *	*	*	*	*	*	*	*	*	*	*	*	*	*	*	*	*	*	*	*	*	*	*	*	*	*	*	*	*	*	*
		b) structured interview *																														
		c) written self report																														
		d) no description																														
	4) Demonstration that outcome of interest was not present at start of study	a) yes *	*	*	*	*	*	*	*	*	*	*	*	*	*	*	*	*	*	*	*	*	*	*	*	*	*	*	*	*	*	*
		b) no																														
Comparability	1) Comparability of cohorts on the basis of the design or analysis	a) study controls for___(select the most important factor) *	*	*	*	*	*	*	*	*	*	*	*	*	*	*	*		*	*	*	*	*	*	*	*		*	*		*	*
		b) study controls for any additional factor ◇_____ (This criteria could be modified to indicate specific _______ control for a second important factor.)*																*									*			*		
Outcome	1) Assessment of outcome	a) independent blind assessment *			*		*																									
		b) record linkage *	*	*		*		*	*	*	*	*			*		*	*	*	*	*	*	*	*	*	*	*	*	*	*	*	*
		c) self report																														
		d) no description																														
	2) Was follow-up long enough for outcomes to occur	a) yes (select an adequate follow up period for outcome of interest) *			*	*	*	*	*	*	*	*	*	*		*	*	*	*	*		*	*	*	*	*		*	*	*	*	
		b) no																														
	3) Adequacy of follow up of cohorts	a) complete follow up - all subjects accounted for *			*	*			*	*	*	*	*	*	*	*	*			*												
		b) subjects lost to follow up unlikely to introduce bias - small number lost - > ____ % (select an___adequate %) follow up, or description provided of those lost) *		*																		*				*				*	*	
		c) follow up rate < ____% (select an adequate %) and no description of those lost																														
		d) no statement																														
Assessment	* Selection (max 4 stars)	*	4	4	4	4	4	4	4	4	4	4	3	4	4	4	4	4	4	4	4	4	4	4	4	4	4	4	4	4	4	4
	* Comparability (max 2 stars)	*	1	1	1	1	1	1	1	1	1	1	1	1	1	1	1	1	1	1	1	1	1	1	1	1	1	1	1	1	1	1
	* Outcome (max of 3 stars)	*	1	2	3	3	2	2	3	3	3	3	2	2	2	2	3	2	2	3	1	3	2	2	2	3	1	2	2	3	3	1
	* Total	*	6	7	8	8	7	7	8	8	8	8	6	7	7	7	8	7	7	8	6	8	7	7	7	8	6	7	7	8	8	6

Note: A study can be awarded a maximum of one star for each numbered item within the Selection and Outcome categories. A maximum of two stars can be given for Comparability
